# Supplementary material for: Students’ figurative communication of malaria messages, belief, norms, and practices in Oromia, Ethiopia: A qualitative content analysis approach
Source: PLoS One. 2022 May 25;17(5):e0268808. doi: 10.1371/journal.pone.0268808 (PMC9132334; doi:10.1371/journal.pone.0268808)
Supplement: Attachment — Submitted filename: Response to reviewer.docx [file pone.0268808.s001.docx]

**Response to reviewers**

**Dear PLOS ONE academic editor and reviewer,**

We want to express our deepest gratitude for reviewing and providing your constructive comments to us on a manuscript entitled “Students’ figurative communication of malaria messages, belief, norms, and practices in Oromia, Ethiopia: A qualitative content analysis approach” submitted to PLOS ONE journal. We are very much grateful for the editor and reviewers’ time and willingness to review the manuscript. The authors found the comments of the editor and reviewers very important. The authors were really very happy to address the comments raised by the editor and reviewer for this manuscript. We have seriously considered the comments and have gone through the entire body of the manuscript to make necessary editorial corrections and clarifications to the concerns. Corrections are shown by highlighting the texts in yellow color in the main document. Please find below our responses based on each comment.

**Response to reviewer 1:**

Thank you very much for your comments. We have amended as per your constructive comments and please find below.

1. Information on the school-based SBCC intervention in the study should be provided: How and why did the students create poems? How were the students educated about malaria?

**Response:** Thank you so much for the comment. The students were used as key actors for the social and behavioral change communication aiming to develop and effectively and efficiently disseminate malaria messages to the wider community to improve the capacity, knowledge, and decision-making skills that help to promote health and prevent diseases. The intervention was conducted in the study setting from 2017 to 2019. At the beginning of the intervention, training was given to the students and focal teachers. Then, under the supervision of the focal teachers, students developed malarial messages in the form of poems and disseminated it to the community through different means. **Introduction, page 3, line 66-85.**

2. The generalizability of the findings should be discussed. This is because the study samples were collected only from the intervention schools.

**Response:** Thank you so much. It is obvious that in qualitative research, the study findings are not generalized to the other settings rather transferred to similar contexts. Therefore, the issue of transferability was discussed under trustworthiness section. **Method, page 6 &7, line 178-172.** In addition, we have described that the poems were selected from schools under the school-based malaria project aimed to advance community knowledge and practices. Thus, the contents of any poems generated out of the project site were not produced. **Strength and Limitation of the Study, page 16, line 473-475**

3. Are there any similar studies to which the authors referred to develop the data analysis strategies and to validate the results? What literature was used for planning the methods?

**Response:** Really, thank you for this comment. We have not yet got studies conducted on similar topics with similar population. However, we used a poetic analysis approach described in different literatures. For example, we have used reference number 18-25 to plan and guide the analysis and method. **Method, page 6, line 150**

4. Writing opinions in the results section should be avoided. For example, in the second paragraph of the “Figurative speeches….malaria”, the authors mentioned “Therefore, anyone who can go through this work should have to understand…”

**Response:** We have amended it as per your comment. **Result, page 8, line 213-219.**
5. It would be better to divide the second paragraph of the discussion section (Simile is…) into two paragraphs, as the paragraph includes two different topics such as anopheles and sign/symptom.

**Response:** We have amended it as per your comment. **Discussion, page 14, line 408.**
6. Importantly, potential risk/negative consequence of “figurative communication” should be discussed. For example, “anopheles mosquito was expressed like…flies” and “malaria was expressed in terms of diseases that is serious than HIV”.

**Response:** Thank you so much for this comment. Yes, it needs some explanation and we have provided the description that it causes misconception and confusion among the audience. **Discussion, page 16, line 466, 469.**

7. In the limitation section, the authors mentioned that “due to limited literature, the findings were not well discussed”. I do not fully agree with the authors, because although there are few studies that examined figurative languages for malaria control, there are a number of studies that examined figurative languages for other topics.

**Response:** Thank you so much. You are right a lot of papers are already available on different topics and it is not mandatory to get similar article. Therefore, we have amended it and used different articles to discuss the findings. **Strength and limitation of the study, page 16, line 471-479**

8. In the conclusion section, the authors stated that “This implies that use of different figurative speeches is very important….”. What does support the statement? Why did the authors consider the figurative speeches very important? Was the conclusion based on the results?

**Response:** It is true that we made a mistake while concluding the study findings. We have amended it to be consistent with the findings. We concluded that the study found that the students conveyed messages related to malarial perceptions, beliefs, norms, and practices of the local community to prevent and control malaria through different types of figurative speeches; simile, metaphor, personification, and hyperbole. Predominantly, the students expressed in their poems that there was malpractice of malaria prevention and control measures, especially on the use of ITN among the community. This underscores that there is a need to design health education programs and conduct SBCC interventions to properly practice malaria prevention and control measures. **Conclusions, page 16, line 481-487**

**Response to reviewer 2:**

Response: Thank you so much for your constructive comments. We accepted all of your comments are valuable enough to improve the quality of the manuscript. Majorly, you have raised the grammatical and typographical errors. Thus, accepted your comments and amended it throughout the manuscript. We have also changed the word “Belief” in the title part to “beliefs”. **Title, page 1, line 1**

2. Important to revisit the paper to improve the grammar which would enhance its readability. An important way to achieve this is replacing the long and winding sentences such as the second paragraph in the introduction section with shorter ones. Similarly, the second sentence could be improved with appropriate punctuation. Consider these for the entire paper.
**Response:** We have amended it as per your constructive comments throughout the manuscript. . 3. The first sentence in the third paragraph should be revisited as well. A poem is not a form of malaria message.

**Response:** Yes, it it true that the poem is not a form of malaria message. We have rephrased it accordingly. **Introduction, page 3, line 82-85**

4. Revisit the second line of the fourth paragraph specifically the sentence “…, and the primary students were developed and disseminated malaria messages…” it does not make sense.

**Response:** We have rephrased it accordingly. **Introduction, page 3, paragraph 3, line 79-82**
5. In the data analysis section, there is content talking about selection of the 20 poems. This information must be removed. The sample and sampling section should have covered this content / information.

**Response:** It is true that this information should be included under sample and sampling technique. Thus, we have removed it from the analysis part. **Method, data analysis, page, 6**
6. As already mentioned, the entire paper should be revisited to improve grammar. In the analysis section, sentences like: “Then, the coding of the whole poems...” should be corrected. Similarly, statement like: “The coding system repeated the four times after the draft code book was developed”. These and various other statements should be revisited.
The data analysis should focus on the specific area of interest (figurative language) that was analyzed than to provide details such as typical analysis done to poems like the structure of the poem and setting of the poem etc. if this did not form part of the eventual themes that are central to this paper.

**Response:** We have accepted your comments and amended all of it. **Method, data analysis, page 6**

7. Adopt a consistent writing / format style. For example, SIMILE and HYPERBOLE are in capitals while Metaphors, in small letters. In this case it seems the main themes were supposed to be in CAPITALS while the sub-themes in small letters. If that is the case, then be consistent.
**Response:** Thank you for your comments again. It is true that we made a mistake while writing it. We have amended it typing as Simile, Metaphor, Personification and Hperbole**. Result, page 8-13, line 221, 260, 345 and 365**

8. The discussion could be discuss more the findings of the study mainly. For example, the opening statements talks about many figurative speeches that the study did not find or work with. While the statement is meant to provide a general picture of figurative speeches that exist, it does not provide a general statement that properly represent the findings of this study.

**Response:** Yes, it is true. Our assumption was to make an introductory sentence rather than indicating all types of figurative speech. Therefore, we have corrected made it consist with the current study findings. **Discussion, page 14, line 397 and 398**

9. The discussion should NOT be a re-presentation of the results. Rather it should interpret and describe the significance of these findings in light of what was already known about the issue / problem being investigated and to explain any new understanding or insights that emerged as a result of this study. This is not coming out clearly. The discussion is largely explaining how the different figurative speeches were used. Ideally it should probably talk about the relevance of using personification in the fight against malaria and linking that with studies that could show the importance of such kind of information. The last paragraph that discuss the hyperbolic expression is a good example of how best to discuss these findings.

**Response:** Thank you again for your comments. We have amended almost all parts of the discussion following your comments. **Discussion, page 14-16.**

10. The study limitation acknowledges the paucity of literature in Ethiopia on this topic and attributes the “inadequate” discussion to this. However, the discussion could use literature from elsewhere if available. Furthermore, even without literature, the discussion could be improved considerably.
**Response:** Thank you so much. You are right a lot of papers are already available on different topics and it is not mandatory to get similar article. Therefore, we have amended it and used different articles to discuss the findings.

11. The conclusion should be revisited. While some elements of what a conclusion is exist, it can be improved. The conclusion should NOT be a summary of the findings, rather it should wrap up the author’s ideas around the topic and leave the reader with a strong final impression of what the study implies.

**Response:** It is true that our conclusion did not fully represent the study findings. However, following your constructive comments, we have revised it and we concluded that the study found that the students conveyed messages related to malarial perceptions, beliefs, norms, and practices of the local community to prevent and control malaria through different types of figurative speeches; simile, metaphor, personification, and hyperbole. Predominantly, the students expressed in their poems that there was malpractice of malaria prevention and control measures, especially on the use of ITN among the community. This underscores that there is a need to design health education programs and conduct SBCC interventions to properly practice malaria prevention and control measures. **Conclusions, page 16, line 481-487**

Saying this, I hope that the comments provided by the reviewer were addressed and the manuscript would meet the high standards of your journal. Therefore, am looking forward to receive a favorable response from you regarding the acceptance of the manuscript.

Sincerely yours

Kasahun Girma Tareke (corresponding author)

Address: Department of Health, Behavior and Society, Faculty of Public Health, Institute of Health, Jimma University, Jimma, Ethiopia

E-mail: [kasahungirmadera@gmail.com](mailto:kasahungirmadera@gmail.com); [girma.tareke@ju,edu.et](mailto:girma.tareke@ju,edu.et);

Phone: +251 919375374
