# Supplementary material for: Students’ figurative communication of malaria messages, belief, norms, and practices in Oromia, Ethiopia: A qualitative content analysis approach
Source: PLoS One. 2022 May 25;17(5):e0268808. doi: 10.1371/journal.pone.0268808 (PMC9132334; doi:10.1371/journal.pone.0268808)
Supplement: Attachment — Submitted filename: Rebuttal Letter.docx [file pone.0268808.s002.docx]

**Rebuttal Letter**

**Dear editor and reviewers,**

Thank you for your constructive comments you provided for us on the manuscript entitled with “Students’ figurative communication of malaria messages, belief, norms, and practices in Oromia, Ethiopia: A qualitative content analysis approach”. We are very much grateful for the editor and reviewers’ time and willingness to review the manuscript. The authors found the comments of the editor and reviewers very important. The authors were really very happy to address the comments raised by the editor and reviewers for this manuscript. We have seriously considered the comments and have gone through the entire body of the manuscript to make necessary editorial corrections and clarifications to the concerns. Corrections are shown by highlighting the texts in yellow color in the main document.

**Response to the Reviewer 3 comments:**

**Comment 1:** Why not quantitative and qualitative study? I think that quantitative study includes large sample size and helps to give detail interpretations about the case? Here you have used purposive sampling techniques, due to this your sample size is very small. If you use random sampling technique, it helps you to study every hidden information about the case.

**Response:** Thank you very much the reviewer. Yes, it was possible to conduct a quantitative study given that it involves large sample size and make the study findings generalized. However, there are issues that demand qualitative study to gain indepth insights about a certain phenomenon. For example, the current study among those health issues that needs contextual interpretation of the figurative speeches or metaphors. Therefor, the nature of our research questions made us to study the pehenomenon using qualitative content analysis approach.

**Comment 2:** You have used purposive sampling, but in your manuscript you are talking about the strata. Why? “This is because they are categorized under the high-medium-malaria-burden strata.” Justify it.

**Response:** Thank you agein for your comment. As it was mentioned in the manuscript, a social and behavioral change communication intervention packages were conducted as selected districs of Jimma zone, oromia, Ethiopia from 2017-2019. The reason why the intervention was conducted at those districts were that malaria endemicity was high and medium, and the intervention aimed to change the behavior of the community through school community engagement.

**Comment 3:** If you are using the purposive sampling technique, what is your reason to select those schools, try to justify it clearly?

**Response**: Thank you again for your noce comments. We accepted as this comment was valuable but forgotten in our manuscript. The schools were selected based on criteria’s such as having high number of students enrolled in the school (ranged from 440-1450), being located at high malaria endemic areas, feasibility in terms of distance and active engagement and better involvement of students in producing poems. **Method section, page 5, line 132-135**

**Comment 4:** In table 1, you have stated the sample allocation, do you think that is it proportional? I am sure that your answer is no, because there is no equal number of poems in each school.

**Response:** Infact, the principle of purposive sampling is doesnot mean or consistent with proportional allocation. This is because is because participant recruitment based on purposive sampling technique depends on the richness of information and criterialss. For example, in the method section, page 5, line 135-143 was mentioned that “A purposive sampling technique was used to select poems from school-based malaria SBCC documentation based on like richness, relevancy and data diversity pertinent to the research question. Specific criteria’s used to select the poems were presence of at least three behavioral constructs (from knowledge, attitude, risk- perception, self-efficacy, response -efficacy, and practice ) through roughly reading; presence and readability of the poems, length of the poems (i.e., at least two pages) and presence at least one type of figurative speech. Accordingly, 20 poems were selected from 20 selected schools across the districts. A sample of poems was selected from different schools across the districts and students grades to maximize data triangulation.” This means that the samples were not proportionally allocated rather determined, by default, by criterials mentioned above.

**Comment 5:** From which class students do you taken the sample, from grade 5, 6, 7 or 8? Because students in each class has different understandings about the case? Justify it.

**Response:** We took sample of poems from all 5-8 classes. Yes. It is true that there might be different level of understanding about the health issue across the grade levels. However, it the **introduction section, page 2, line 72-80 of the manuscript, it** was mentioned that adequate training was given to all students from 5-8 grade levels to made their understanding close enough as much as possible.

**Comment 6:** What does it mean that “Note: Equal of 5 poems will be considered from grade 5, 6, 7 and 8” in table 1. Explain it clearly.

**Response:** Thank you for the comment. It was to mean five poems were purposively selected from grade 5, 6, 7 and 8. **Method section, page 5, table 1**

**Comment 7:** Do you believe that this sample is the representative of the Jimma Zone primary school students, or can you conclude about the cases in Jimma zone based on this sample?

**Response:** Thank you gain for the comment. Infact, in the paradigm of quantitative research the sample size was very small to represent the sstudy population and mage the study finding generalize. However, in case of qualitative research, this is different. The nature of qualitative research is to recruit small sample size from different contects (setting, population, time, etc). Therefore, in this study, poems prepared by students from grade 5, 6, 7 and 8 were analyzed. In addition, the poems were selected from different schools or districs. Therefore, only sample size doesnot deteremine representativeness in qualitative research. Rather thickly describing the research process and contexts ensure dependability and transferability of the findings. **These issues were described in the manuscript, under trustworthiness section, page 6 and 7, line 176-201.**

**Comment 8:** In you result part you have some descriptive statistics “The ages of students who developed the poems ranged from 12 to17 years old (mean age, 14.3 years). Females and males each contributed ten poems.” But, it is not presented in the tabular form, try to present it in tabular form. Not only this, try to put all results that you have collected like age of students and sex of students.

**Response:** Thank you so much! We accepted as it was and amended it accordingly. **Result section, page 8, Table 2**

**Comment 9:** In general, you missed study designing and sampling techniques in your manuscript revise it seriously.

**Response:** It is obvious that qualitative study has different approaches. Different schoolars donot use the term ‘study design’ is qualitative study rather’study approaches’. This is because it fouses on an indepth or insight interpretation of meanings. Therefore, we used a qualitative content analysis approach to interprete figururative speeches used in the poems. **Method section, page 4, line 122-127.**

Saying this, we hope that the comments provided by the reviewer were addressed and the manuscript would meet the high standards of your journal. Therefore, we are looking forward to receive a favorable response from you regarding the acceptance of the manuscript.

Sincerely yours

Kasahun Girma Tareke (corresponding author)

Address: Department of Health, Behavior and Society, Faculty of Public Health, Institute of Health, Jimma University, Jimma, Ethiopia

E-mail: [kasahungirmadera@gmail.com](mailto:kasahungirmadera@gmail.com); [girma.tareke@ju,edu.et](mailto:girma.tareke@ju,edu.et);

Phone: +251 919375374

**Thank you!!!**
